# Promotion of a venous thromboembolism prevention protocol at a perioperative management center

**DOI:** 10.20407/fmj.2022-024

**Published:** 2022-12-27

**Authors:** Himuro Fujiwara, Chizuru Yamashita, Takahiro Kawaji, Tomoyuki Nakamura, Naohide Kuriyama, Yoshitaka Hara, Satoshi Komatsu, Minako Fukushima, Shinya Suzuki, Takaaki Tsutsumi, Osamu Nishida

**Affiliations:** Department of Anesthesiology and Critical Care Medicine, Fujita Health University, School of Medicine, Toyoake, Aichi, Japan

**Keywords:** Anticoagulant, Perioperative management center, Venous thromboembolism, Lower-limb compression ultrasonography, Secondary thromboprophylaxis

## Abstract

**Objectives:**

Perioperative venous thromboembolism (VTE) is a potentially fatal complication, making preoperative VTE diagnosis and secondary thromboprophylaxis important. This study was performed to investigate the impact of promotion of a preoperative VTE protocol at a perioperative management center (PMC) on detecting the preoperative VTE rate and subsequent treatment.

**Methods:**

This retrospective study involved patients aged ≥20 years who underwent elective anesthesia. The patients were divided into two groups: the pre-PMC group (January to October 2014, before the opening of the PMC) and the post-PMC group (January to December 2019, after the opening of the PMC). The rates of preoperative lower-limb compression ultrasonography (CUS), VTE detection, anticoagulation therapy, and new postoperative pulmonary embolism (PE) were compared between the two groups.

**Results:**

The pre-PMC and post-PMC groups comprised 3737 and 5388 patients, respectively. The preoperative CUS and VTE detection rates were significantly higher in the post-PMC than pre-PMC group (7.2% and 1.43% vs. 25.6% and 3.93%, respectively; P<0.001). There was no significant difference in the rate of anticoagulation therapy in patients with preoperative VTE (88.9% vs. 84.7%, P=0.43). Heparin and direct oral anticoagulants were primarily used in the pre-PMC and post-PMC groups, respectively. The efficacy and safety were comparable between the two groups. No new postoperative PE was detected in either group.

**Conclusions:**

Promotion of the preoperative VTE protocol led by the PMC increased the rates of preoperative CUS and preoperative VTE detection. This may aid in secondary thromboprophylaxis in the preoperative period and prevention of postoperative PE.

## Introduction

The purpose of a perioperative management center (PMC) is to provide a safe perioperative environment for patients undergoing surgery. Perioperative pulmonary embolism (PE) is a highly lethal but preventable healthcare-related complication. Surgical invasion is associated with all three factors of thrombosis (vascular injury, venous stasis, and increased coagulability), and patients have a high risk of developing venous thromboembolism (VTE) in the perioperative period.^1,2^ Moreover, complications of malignancy and prolonged perioperative bed rest further increase the risk.^3,4^ Studies have also shown that patients with preoperative deep vein thrombosis (DVT) are at high risk of peripheral to central thrombosis if anticoagulation and physical prophylaxis are not administered in the perioperative period.^5,6^ Therefore, in patients whose condition is complicated by preoperative VTE, detection of preoperative VTE and prevention of secondary thrombosis are important to prevent PE in the perioperative period. However, there are no specific guidelines for the prevention of secondary thrombosis.

**Figure 1:**
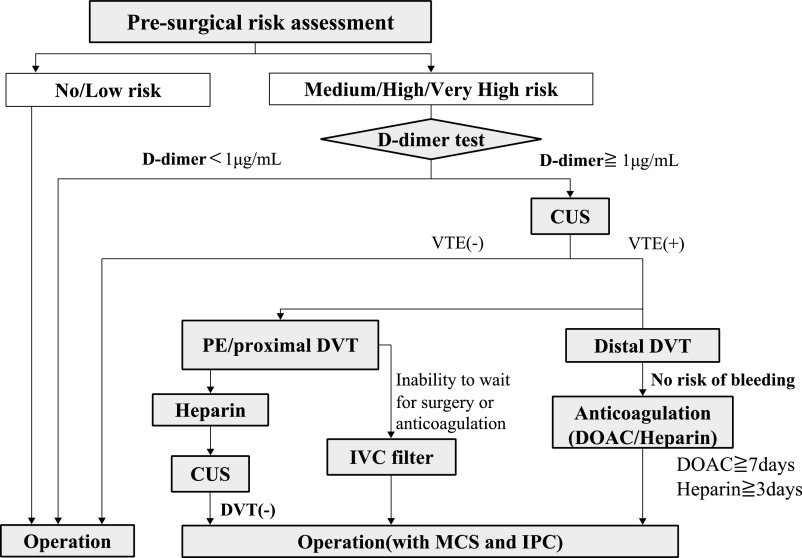
Preoperative VTE prevention and treatment protocol The preoperative VTE protocol first evaluates the patient’s risk of preoperative VTE on a five-level scale according to the patient’s characteristics and procedure. Second, patients at intermediate risk or higher are screened by measurement of their D-dimer level, and if the D-dimer level is ≥1.0 μg/mL, VTE is diagnosed by CUS. If peripheral DVT is detected, anticoagulation with DOAC or heparin is administered with consideration of the period until surgery. If central DVT or pulmonary thrombosis is detected, anticoagulation with heparin is initiated, and surgery is performed after thrombus resolution. CUS, lower-limb compression ultrasonography; VTE, venous thromboembolism; DVT, deep vein thrombosis; IVC, inferior vena cava; DOAC, direct oral anticoagulant; MCS, medical compression stockings; IPC, intermittent pneumatic compression

At Fujita Health University Hospital, we have been actively preventing preoperative secondary thrombosis using a specific protocol since 2013 (Figure 1). Initially, decisions regarding preoperative testing and treatment were primarily made by attending physicians. Furthermore, the anesthesiologist performed a VTE risk assessment immediately before surgery, leaving no time for preoperative intervention by anesthesiologists. After establishing the PMC in 2015, patients began visiting the center 2 to 4 weeks before their elective surgery, allowing anesthesiologists to be actively involved in implementation of the protocol.

We hypothesized that implementation of our preoperative VTE protocol centered at the PMC would be effective in increasing the detection rate of preoperative VTE and promoting preoperative secondary thromboprophylaxis. We therefore compared the results before and after establishing the PMC.

## Methods

### Research design

This study was conducted after receiving approval from the institutional review board of Fujita Health University (HM20-316), and the patients’ medical records were retrospectively reviewed. The participants were patients aged ≥20 years who underwent elective general anesthesia. The patients were divided into two groups: the pre-PMC group (from January to October 2014, before establishment of the PMC) and the post-PMC group (from January to December 2019, after establishment of the PMC). VTE risk assessment and protocol operations were performed mainly at the attending physician’s discretion in the pre-PMC group, and the anesthesiologists checked the patients 1 day before surgery. In contrast, the patients in the post-PMC group visited the PMC 2 to 4 weeks before surgery, at which time the anesthesiologists confirmed the VTE risk and were involved in the protocol-based procedure as needed. VTE risk assessment was based on three levels of risk according to the procedure (low, medium, and high risk) plus any additional risk the patient had, resulting in a final risk classification of five levels (no, low, medium, high, and very high risk) (Figure 2). The D-dimer concentration was measured when the final VTE risk was assessed as medium or higher according to the protocol. Patients with high D-dimer levels (≥1.0 μg/mL) underwent lower-limb compression ultrasonography (CUS), in which the vein was compressed with a probe to check for the presence of a thrombus according to the compressibility of the vein. The common femoral vein was evaluated along with the tibial, fibular, and soleal veins. A computed tomography scan was performed if extension of the thrombus beyond the common femoral vein into the pelvis inferior vena cava, or pulmonary artery was suspected. Thrombus sites were classified into four categories: lower leg, thigh, pelvis and inferior vena cava, and pulmonary artery. Thrombi that were noted at more than one site simultaneously were classified as centrally located. For the lower leg, bilateral and unilateral classifications were performed. If VTE was detected, anticoagulation therapy was administered at the attending physician’s discretion in the pre-PMC group. Anesthesiologists were also included in the post-PMC group.

### Items for consideration

The patients’ backgrounds (age, sex, body mass index, and department in which treatment was performed) and the number of preoperative VTE tests (CUS and computed tomography) were examined in this study. In the post-PMC group, the protocol compliance rate (percentage of tests performed according to the preoperative VTE protocol) was calculated. The preoperative VTE detection rate and percentage of thrombus sites were examined to determine the detection status of preoperative VTE. The number and rate of perioperative compression treatments for preoperative VTE-positive patients were calculated. The number of patients receiving preoperative anticoagulation and the type of anticoagulant used were reviewed to determine the preoperative anticoagulation status. We also examined the development of a new postoperative PE as an outcome of preoperative anticoagulation therapy. The number of thrombus changes (disappearance, shrinkage, enlargement, or appearance of a new thrombus) was investigated in patients who underwent postoperative CUS thrombus evaluation. Efficacy of anticoagulation therapy was defined as disappearance or shrinkage of the thrombus, and the efficacy rate was calculated. The valid period for postoperative CUS examination was defined as the period within 1 month of surgery. Moreover, in patients with preoperative VTE in the post-PMC group, we examined the relationship between the D-dimer level and the number and detection rate of preoperative VTE and the relationship between age and the detection rate of preoperative VTE.

### Statistical analysis

All analyses were performed using the statistical software EZR Ver. 1.55 (Saitama Medical Center, Jichi Medical University, Saitama, Japan). All data are expressed as median (interquartile range) and were tested using the chi-square test, Fisher’s exact test, and the t-test. A P value of <0.05 was considered statistically significant.

## Results

### Patient characteristics and number of preoperative VTE tests

The pre-PMC group comprised 3737 patients, and the post-PMC group comprised 5388 patients. Table 1 shows the patient characteristics and number of preoperative VTE tests performed according to the preoperative VTE protocol. The patients’ median age in the pre-PMC and post-PMC groups was 63 and 64 years, respectively. In terms of the department, general gastroenterological surgery was the most common department in both the pre-PMC and post-PMC groups, accounting for 21.8% and 18.9% of the total, respectively. Orthopedic surgery was the next most common department, accounting for 9.7% and 11.3% of the cases, respectively. Preoperative CUS was performed in 270 (7.2%) and 1379 (25.6%) patients in the pre-PMC and post-PMC groups, respectively (P<0.001). Protocol compliance in the post-PMC group was 96.9%.

### Preoperative VTE detection status

The detection rates of preoperative VTE are shown in Table 2. Preoperative VTE was detected in 54 (1.42%) patients in the pre-PMC group. In contrast, preoperative VTE was detected in 209 (3.93%) patients in the post-PMC group, with a statistically significant difference (P<0.001). The most common thrombus site was the lower leg in both groups. There was no significant difference in the percentage of thrombus sites between the two groups. Furthermore, preoperative asymptomatic PE was detected in one patient in the pre-PMC group and in three patients in the post-PMC group, all of whom had DVT in their lower legs.

### Perioperative compression treatment

Intraoperative medical compression stockings were used in 46 (85.2%) patients in the pre-PMC group and in 201 (96.2%) in the post-PMC group (P=0.007). Intraoperative intermittent pneumatic compression (IPC) was performed in 36 (66.7%) patients in the pre-PMC group and in 167 (79.9%) in the post-PMC group (P=0.046). Patients who did not receive compression treatment included those who underwent simultaneous surgery on both lower limbs and those who had pressure ulcers on the lower limbs, among others.

### Preoperative anticoagulation and outcomes of anticoagulation therapy

The status of anticoagulation therapy is shown in Table 3. Among the patients with preoperative VTE, 48 (88.9%) and 177 (84.7%) in the pre-PMC and post-PMC groups received anticoagulation therapy (P=0.43). Regarding the type of anticoagulant, heparin was used in 97.9% of patients in the pre-PMC group; in contrast, direct oral anticoagulants (DOACs) were the most common anticoagulant in the post-PMC group (66.1%), and heparin was used in 32.2%.

No new cases of postoperative PE occurred in either group. Using CUS, postoperative thrombus evaluation was performed in 20 and 19 patients in the pre-PMC and post-PMC groups, respectively (Table 4). The efficacy rate (disappearance or shrinkage of the thrombus) of preoperative anticoagulation therapy was 60.0% and 68.4% in the pre-PMC and post-PMC groups, respectively, with no significant difference between the two groups.

### Relationship between D-dimer level and number and detection rate of preoperative VTEs and relationship between age and detection rate of preoperative VTE

**Figure 3:**
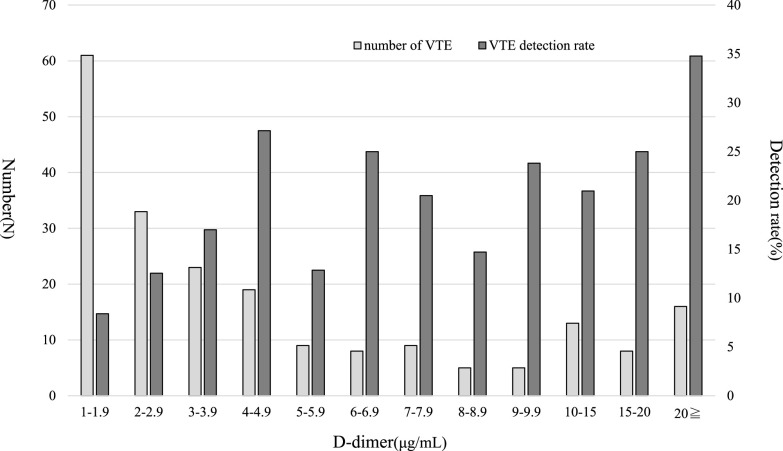
Determination of number and rate of preoperative VTEs by D-dimer range in post-PMC group The number and rate of preoperative VTEs in the post-PMC group according to the D-dimer range are shown. The highest number of preoperative VTEs was detected in patients with a D-dimer range of 1.0 to 1.9 μg/mL. The rate of preoperative VTE was lowest in the same range and highest in the D-dimer range of ≥20 μg/mL. VTE, venous thromboembolism; PMC, perioperative management center.

**Figure 4:**
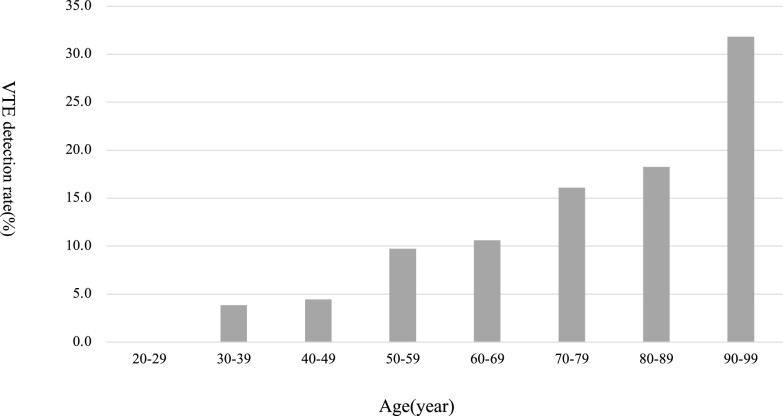
Age-specific VTE detection rate in post-PMC group The preoperative VTE detection rates according to age in the post-PMC group are shown. The preoperative VTE detection rate tended to increase with age. VTE, venous thromboembolism; PMC, perioperative management center.

Figure 3 shows the number and detection rate of preoperative VTEs by D-dimer level in patients with preoperative VTE complications in the post-PMC group. The highest number of preoperative VTEs was detected in 61 patients with a D-dimer range of 1.0 to 1.9 μg/mL. Meanwhile, the preoperative VTE detection rate was lowest at 8.4% for patients with a D-dimer range of 1.0 to 1.9 μg/mL and highest in patients with a D-dimer level of ≥20 μg/mL. The relationship between age and the preoperative VTE detection rate is shown in Figure 4. The preoperative VTE detection rate was 3.8% in patients aged 30 to 39 years and 31.8% in patients aged ≥90 years, showing an increasing trend with age.

## Discussion

This study showed that promotion of the preoperative VTE protocol led by the PMC increased the rate of preoperative CUS and the rate of preoperative VTE detection. Secondary thrombus formation in patients with preoperative DVT can result in floating thrombi and PE. In a report of patients with femoral neck fractures, all patients who developed PE in the perioperative period had thrombi in the intramuscular venous plexus of the soleus and/or gastrocnemius muscles.^7^ In the present study, all patients in whom preoperative asymptomatic PE was detected also had lower-leg DVT complications. Thus, even lower-leg DVT can be a serious risk factor for PE in the perioperative period. Therefore, preoperative screening and diagnosis of VTE are important for preventing perioperative PE.

Our preoperative VTE protocol consisted mainly of D-dimer measurement and CUS. The CUS rate significantly increased from 7.2% before establishment of the PMC to 25.6% after its establishment. There were two reasons for this increase. First, the anesthesiologists, playing a central role in the PMC, followed the protocol and performed a thorough preoperative risk assessment. The protocol compliance rate in the post-PMC group was high (96.9%). Second, patients were seen at the PMC 2 to 4 weeks before surgery, allowing more time to perform additional tests. The increase in the rate of preoperative VTE detection may be due largely to the increase in the rate of preoperative CUS.

Intraoperative compression treatment with medical compression stockings and IPC were performed at a high rate according to our protocol in both the pre-PMC and post-PMC groups. The use of intraoperative IPC in patients with DVT is controversial. A patient with DVT in the proximal veins of the bilateral lower limbs reportedly developed PE after induction of general anesthesia while using IPC.^8^ In contrast, studies have shown that compression treatment does not increase the risk of PE and is useful in improving the clinical presentation of DVT.^9^ Our protocol also prohibits the use of IPC in patients with large DVT or free DVT. No patients developed intraoperative PE in this study, suggesting that IPC can be safely used at least for distal type DVT.

From both prophylactic and therapeutic perspectives, anticoagulation is important in the perioperative management of VTE. Perioperative PE is difficult to prevent using mechanical prophylaxis alone,^10^ and non-use of anticoagulants is a prognostic factor for perioperative PE.^11^ In this study, the rate of preoperative anticoagulation among patients in whom preoperative VTE was detected was not different between the pre-PMC and post-PMC groups. However, the number of preoperative anticoagulation procedures increased as the preoperative VTE detection rate increased. Moreover, heparin was used in most patients with preoperative VTE in the pre-PMC group, but DOAC use surpassed heparin use in the post-PMC group because DOACs were approved for VTE in September 2014. DOACs have comparable antithrombotic effects and a lower bleeding risk when compared with warfarin^12–14^ and may be safe for use in the perioperative period. Another advantage is that DOACs have a rapid onset of action after oral administration and can be prescribed without hospitalization.^15^

There was no difference in the outcome of anticoagulation therapy between the two groups, with no new cases of postoperative PE in either group. The low incidence of PE and insufficient sample size to compare PE rates may be the main reasons for the lack of difference in this outcome. We believe that the lack of difference between the two groups does not negate the benefit of anticoagulation in patients with preoperative VTE. There was no difference in the availability ratio of preoperative anticoagulation in patients who underwent postoperative CUS between the two groups. The protocol did not include postoperative follow-up for VTE, which may have contributed to the low number of cases in which CUS was performed. In particular, cancer-related VTE has a higher incidence and risk of recurrence than non-cancer-related VTE and necessitates longer-than-usual anticoagulation therapy.^16^ In future, it would be desirable to improve compliance with preoperative VTE protocols while considering the bleeding risk in individual patients and further promote postoperative anticoagulation therapy and CUS follow-up.

Age, comorbidities (e.g., cancer, aortic disease, and fractures), and other factors all influence the D-dimer level, and perioperative patients frequently have more than one of these factors.^17^ Some reports have suggested that the D-dimer cutoff should be increased by adjusting for age in patients aged >50 years.^18–20^ Because measurement of the D-dimer level is said to be highly specific for thrombi, we set the cutoff D-dimer level in the preoperative VTE protocol at 1.0 μg/mL, which is the upper limit of the reference range at our institution. In the analysis of preoperative VTE by the D-dimer range, VTE detection at 1.0 to 1.9 μg/mL was the most common, with detection occurring in 61 (29%) patients, although the rate was slightly lower than the other D-dimer ranges at 8.4%. Assuming that age-corrected D-dimer cutoffs were used in the study results, 62.7% of patients with a D-dimer level ranging from 1.0 to 1.9 μg/mL in whom preoperative VTE was detected had a D-dimer level below the cutoff, resulting in undiagnosed VTE. Some reports suggest that the cutoff D-dimer level should be rather low for cancer-related VTE.^21^ However, a lower cutoff level leads to a lower CUS-positive detection rate. Therefore, further studies regarding the evaluation of D-dimer cutoff values are warranted.

This study had two main limitations. First, the incidence of perioperative PE is low, ranging from 0.10% to 0.23%,^22^ and the sample size was therefore considered insufficient to compare the incidence of PE as an outcome. Second, postoperative CUS follow-up was not incorporated into the protocol and was instead performed at the attending physician’s discretion, which may have introduced selection bias in the study of anticoagulation efficiency.

In conclusion, promotion of the preoperative VTE protocol led by the PMC increased the rate of preoperative CUS performed and the rate of preoperative VTE detection, which may have led to the promotion of preoperative secondary thromboprophylaxis. Perioperative PE is considered a preventable complication, and anesthesiologists need to play a central role in the PMC to provide a safer perioperative environment.

## Figures and Tables

**Figure 2:** VTE risk assessment: procedure-specific risk and additional risk The risk of VTE is classified into three levels by department and procedure, and then into five final risk levels (i.e., no, low, medium, high, and very high risk of VTE) based on the patient’s total score of additional risk. If the total score is –2 points, the risk is lowered by one level. If the total score is –1 to +1 point, there is no change. If the total score is +2 to 3 points, the risk is increased by one step. If the total score is +4 to 6 points, the risk is increased by two steps. Finally, if the total score is +7 or more points, the risk is increased by three steps. VTE, venous thromboembolism; BMI, body mass index.

Figure 2-1			
Department	Low risk	Medium risk	High risk
Departments except orthopedics, neurosurgery, obstetrics	• Non-major surgery under 60 years • Major surgery under 40 years	• Non-major surgery over 60 years or having risk factors • Major surgery over 40 years or having risk factors	• Major surgery for cancer over 40 years

Orthopedics	• Upper limb surgery	• Upper limb surgery involving bone extraction from the ilium or nerve and skin extraction from the lower limb • Spinal surgery • Lower limb surgery	• Artificial hip or knee joint surgery • Hip or femoral diaphyseal fracture surgery • Pelvic osteotomy (Chiari pelvic osteotomy, rotational acetabular osteotomy, etc.) • Lower limb malignancy surgery • Pelvic fracture

Neurosurgery	• Surgery other than craniotomy	• Craniotomy other than brain tumor	• Craniotomy for brain tumor

Obstetrics	• Normal delivery	• Caesarean section (other than high risk)	• Cesarean section in older obese pregnancy

Trauma		• Spine and spinal cord injury • Single trauma below the distal femur	• Severe trauma (multiple trauma) • Severe burns • Severe spinal injury (cervical spine injury)

Figure 2-2					
Additional risk	• Under 40 years (except orthopedics, neurosurgery, obstetrics)	• BMI>25 • Laparoscopic surgery • Estrogen therapy • Leg vein varicose	• Over 60 years (except orthopedics, neurosurgery) • Bed rest for 48 hours or more • Congestive heart failure • Respiratory failure • Central venous catheter placement • Chemotherapy • Severe infections • Severe leg vein varicose	• Lower limb paralysis • Traction • Bandage immobilization with cast of lower limb	• Previous VTE • Thrombophilia (antithrombin deficiency, protein C deficiency, protein S deficiency, and antiphospholipid syndrome, etc.)

Score	−2 points	+1 points each	+2 points each	+3 points each	+9 points each

The final risk is derived from the additional risk added to the procedure-specific risk.

**Table1:** Patient characteristics

	pre-PMC group (n=3737)	post-PMC group (n=5388)	p value
Age, years	63 (46–72)	64 (49–73)	**<0.001**
Sex			
Male, n (%)	1564 (41.9)	2449 (45.6)	**0.001**
Female, n (%)	2173 (58.1)	2939 (54.5)	
BMI, kg/m^2^	22.2 (19.9–24.7)	22.8 (20.4–25.3)	**<0.001**
Department			
General gastroenterological surgery, n (%)	815 (21.8)	1019 (18.9)	**0.001**
Orthopedic surgery, n (%)	493 (13.2)	979 (18.2)	**<0.001**
Head and neck surgery, n (%)	365 (9.8)	472 (8.8)	0.103
Thoracic surgery, n (%)	364 (9.7)	611 (11.3)	**0.02**
Obstetrics and gynecology, n (%)	337 (9.0)	426 (7.9)	0.066
General surgery, n (%)	331 (8.9)	453 (8.4)	0.3
Neurosurgery, n (%)	313 (8.4)	364 (6.8)	**0.006**
Urology, n (%)	191 (5.1)	312 (5.8)	0.149
Plastic surgery, n (%)	104 (2.8)	146 (2.7)	0.39
Other, n (%)	54 (1.4)	72 (1.5)	0.363
Preoperative CUS, n (%)	270 (7.2)	1373 (25.5)	**<0.001**
Preoperative CT, n (%)	0	1 (0.02)	1
Preoperative CUS+CT, n (%)	1 (0.03)	6 (0.11)	0.252

BMI: body mass index, CUS: compression ultrasonography, CT: computed tomography P-values by department are based on residual analysis

**Table2:** Preoperative VTE detection and thrombus site

	pre-PMC group (n=3737)	post-PMC group (n=5388)	p value
VTE (+), n (%)	54 (1.4%)	209 (3.9%)	**<0.001**
Thrombus sites			
Lower leg, n (%)	48 (88.9%)	187 (89.5%)	1
Unilateral, n (%)	35 (64.8%)	142 (67.9%)	0.745
Bilateral, n (%)	13 (24.1%)	45 (21.5%)	0.714
Thigh, n (%)	5 (9.3%)	17 (8.1%)	0.785
Pelvic+IVC, n (%)	0	2 (1.0%)	1
Pulmonary artery, n (%)	1 (1.9%)	3 (1.4%)	1

VTE: venous thromboembolism, IVC: inferior vena cava

**Table3:** Preoperative anticoagulation therapy

	pre-PMC group (n=54)	post-PMC group (n=209)	p value
Preoperative anticoagulation (+), n (%)	48 (88.9%)	177 (84.7%)	0.43
Heparin, n (%)	47 (97.9%)	57 (32.2%)	
Fondaparinux, n (%)	1 (2.1%)	0	
DOAC, n (%)	0	117 (66.1%)	
Warfarin, n (%)	0	2 (1.1%)	
Cilostazol, n (%)	0	1 (0.6%)	

DOAC: direct oral anticoagulant

**Table4:** Outcomes of preoperative anticoagulation therapy by postoperative CUS follow-up

	pre-PMC group (n=20)	post-PMC group (n=19)	p value
Disappearance, n (%)	9 (45.0%)	10 (52.6%)	0.752
Shrinkage, n (%)	3 (15.0%)	3 (15.8%)	1
No change, n (%)	5 (25.0%)	4 (21.1%)	1
Enlargement or new thrombi, n (%)	3 (15.0%)	2 (10.5%)	1

CUS: compression ultrasonography
